# The influence of haemodialysis on CD4+ T-cell counts in people living with human immunodeficiency virus with end-stage kidney disease

**DOI:** 10.4102/sajhivmed.v21i1.1125

**Published:** 2020-12-21

**Authors:** Melanie Pretorius, Estee Benade, June Fabian, Denise Lawrie, Elizabeth S. Mayne

**Affiliations:** 1 Department of Molecular Medicine and Haematology, Faculty of Health Sciences, University of the Witwatersrand, Johannesburg, South Africa; 2 National Health Laboratory Services, Johannesburg, South Africa; 3 Charlotte Maxeke Johannesburg Academic Hospital, Johannesburg, South Africa; 4 Department of Laboratory Medicine, Saskatchewan Health Authority, Regina, Saskatchewan, Canada; 5 School of Clinical Medicine, Faculty of Health Sciences, Wits Donald Gordon Medical Centre, University of the Witwatersrand, Johannesburg, South Africa; 6 Department of Immunology, Faculty of Health Sciences, University of the Witwatersrand, Johannesburg, South Africa

**Keywords:** CD4 T-cell count, hemodilaysis, transplantation, infectious diseases, HIV

## Abstract

**Background:**

In South Africa it is estimated that 7.9 million people are living with human immunodeficiency virus (HIV). HIV is associated with an increased risk of kidney disease. For people living with HIV (PLWH) who develop end-stage kidney disease (ESKD), access to renal replacement therapy can be difficult. Kidney transplantation is a cost-effective option, with improved overall survival and better quality of life. In Johannesburg, the eligibility criteria for kidney transplantation include a sustained CD4+ T-cell count of > 200 cells/μL and suppressed HIV replication.

**Objective:**

To investigate the influence of haemodialysis on the lymphocyte subsets in PLWH with ESKD. In addition, all available %CD4+ T-cell counts, absolute CD4+ T-cell counts and viral load measurements were collected to assess the longitudinal trends of these measurements in PLWH with ESKD.

**Methods:**

This was a cross-sectional study comparing two groups. The HIV-infected study participants (*n* = 17) and HIV-uninfected controls (*n* = 17) were recruited from renal dialysis centres in Johannesburg from 2017 to 2018. Demographic data and social data were collected from all the study participants (*n* = 17). Blood samples were collected from all the study participants (before and after a haemodialysis session), and the lymphocyte subsets were then measured. The available longitudinal data for the serial CD4+ T-cell counts and HIV viral loads were collected (*n* = 14).

**Results:**

Our cohort showed a statistically significant increase in the post-dialysis percentage of CD4+ T cells (5%, *p* < 0.001) and the absolute CD4+ T-cell counts (21 cells/µL, *p* < 0.03). The longitudinal trend analysis for the percentage of CD4+ T cells revealed a significant increase in five participants (36%), and a single patient (7%) had a significant decrease in the longitudinal trend analysis for the absolute CD4+ T-cell counts. The longitudinal trend analysis for HIV viral load revealed the majority of our participants were not virologically suppressed.

**Conclusion:**

This study showed that haemodialysis does not have an immediate negative impact on CD4+ T-cell count, suggesting that immunologic recovery is not impeded by treatment of the underlying ESKD.

## Context

In South Africa, human immunodeficiency virus (HIV) infection remains a leading healthcare concern. In 2016, it was estimated that approximately 7 million people are infected (according to STATS SA). Of these 7 million HIV-infected patients, only 70.7% (~5.3 million) of patients are currently receiving combined antiretroviral therapy (ART).^1^

HIV infection leads to widespread immunological and subsequent organ dysfunction. End-stage kidney disease (ESKD) in HIV infection has been attributed to a number of causes (Table 1) including HIV-mediated renal damage, exposure to nephrotoxic agents including tenofovir disoproxil fumarate and the presence of opportunistic infections. In patients on ART, with a reduction in opportunistic infections, there is a concomitant increased prevalence of non-communicable diseases including diabetes mellitus and hypertension.^2,3,4^

**TABLE 1:** Causes of renal dysfunction in people living with human immunodeficiency virus.

Variable	Description
**Acute kidney injury**^2,3,4^	Dehydration secondary to gastroenteritis Sepsis and opportunistic infections (e.g. *Mycobacterium tuberculosis*) HIV-associated thrombotic microangiopathies (e.g. TTP/HUS)
**Chronic kidney disease**^2,3,4^	Glomerular lesions HIV-associated nephropathy (HIVAN) HIV-associated nephropathy with focal glomerulosclerosis (HIV-FSGS) HIV-immune complex deposition (HIVICD) Other glomerulonephropathies (including amyloidosis, minimal change disease, immunotactoid nephropathy) Tubulointerstitial disease Proximal tubular injury – tenofovir toxicity Chronic tubular injury – amphotericin, tenofovir toxicity Crystal nephropathy – ciprofloxacin, Acyclovir (intravenous) Interstitial nephritis – infections (hepatitis B), immune reconstitution inflammatory syndrome following ART.
**Comorbid diseases**^2,3,4^	Hypertensive nephrosclerosis Diabetic nephropathy Autoimmune disease (lupus nephritis)
**Genetic predisposition**^2,3,4^	Apolipoprotein-1 (APOL1) genetic variants

TTP, thrombotic thrombocytopenia purpura; HUS, hemolytic uraemic syndrome; ART, antiretroviral therapy.

Renal replacement therapy (RRT) for patients with ESKD comprises two modalities – kidney transplantation and chronic dialysis therapy, which can be either haemodialysis or peritoneal dialysis.

Chronic dialysis therapy is expensive for multiple reasons. At a health system level, the provision of chronic dialysis services requires highly trained medical professionals, expensive equipment that needs maintenance, high-volume consumables, water purification systems (for haemodialysis) and a dedicated space for dialysis that has access to in-hospital services.^5^ For the individual with ESKD, chronic dialysis requires regular monitoring of critical indices with blood tests, expensive pharmacotherapeutics such as parenteral iron and erythropoietin and creatinine and ongoing patency of access for dialysis, either with a peritoneal catheter or with vascular access for haemodialysis. This adds substantial cost for healthcare providers (whether state or private) and, when not funded, can be passed on to individuals as ‘out-of-pocket’ expenses. These dialysis-related expenses occur in addition to the costs of treating additional comorbidities such as hypertension, diabetes and HIV infection.^6^

Limited haemodialysis slots are available for patients with ESKD.^7^ In South Africa, access to RRT is disparate, with 189 slots for renal dialysis per million population overall, but only 71.9 per million population available to the public sector.^7^ Currently no national policy is available regulating access to RRT in South Africa. A recent audit conducted in the Western Cape revealed that, of all the patients receiving dialysis, only 10% were people living with HIV (PLWH).^8^ In view of these limitations, kidney transplantation is an attractive option. Kidney transplantation is a curative therapy that prolongs life in patients with ESKD and is more cost-effective even in complicated cases with high levels of sensitisation.^9,10^

Kidney transplantation in PLWH has shown improved overall survival outcomes when compared to PLWH on chronic haemodialysis.^11^ Morbidity and mortality data also suggest that outcomes after renal transplantation are similar in HIV-infected and -uninfected patients.^12,13^ In South Africa, HIV infection was previously considered a contraindication for both chronic haemodialysis and renal transplantation, but this policy has been revised (after 2009).^14^ This is in line with regulations internationally including the 2013 United States HIV Organ Policy Equity Act.^15^ This law also authorised the use of HIV-infected organs for transplantation in PLWH. In South Africa, the outcomes in PLWH undergoing kidney transplantation are equivalent to those seen in other studies for both HIV-infected and -uninfected donor pools. Some centres in South Africa have begun utilising organs from HIV-infected deceased donors, with 100% 1-year graft survival.^14^

The Wits Donald Gordon Kidney Transplant programme is one of the largest national programmes. Listing of PLWH as recipients commenced in Johannesburg in 2014. The current guidelines for eligibility for deceased-donor kidney transplantation in an HIV-infected individual in the Johannesburg transplant program include stable ART with good adherence for the past 6 months, absence of acquired immunodeficiency syndrome (AIDS)-defining illnesses, CD4+ T-cell counts of > 200 cells/µL for 6 months and undetectable viral load for more than 6 months.^16^

The CD4+ T-cell count is an important risk predictor of patients undergoing transplantation. Patients with absolute CD4+ T-cell count of < 200 cells/µL are at an increased risk of opportunistic infections, have a higher post-transplant rejection rate and present with delayed CD4+ T-cell count recovery after the procedure.^17^ Although HIV infection is the primary driver of the reduced CD4+ T-cell count in PLWH, other factors may also impact the peri-transplant immune status of patients including the use of chronic haemodialysis. Previous studies, examining the impact of haemodialysis on leucocyte counts and leucocyte subsets, have been performed in the past on HIV-uninfected cohorts. The findings of these studies are contradictory. Generally, they showed consistently decreased levels of CD3+, CD4+ and CD8+ T cells. However, these measurements were taken at various intervals between haemodialysis and not immediately post-dialysis.^11^ These studies postulated that direct contact between lymphocytes and dialyser membranes could result in activation of lymphocytes with subsequent apoptosis.^11,12^

A concern, therefore, exists that chronic haemodialysis could reduce CD4+ T-cell count, especially in PLWH, and this would impact their eligibility for the deceased donor list. The aim of this study was to measure immediate and ongoing T-cell counts and T-cell subsets to evaluate the immediate influence of haemodialysis on the lymphocyte subsets in PLWH having ESKD receiving chronic haemodialysis.

## Design

This was a cross-sectional study that compared two groups at the same time. The study participants (*n* = 17) included all eligible HIV-infected adults with ESKD receiving chronic haemodialysis (three sessions a week, each lasting ~4 hours), irrespective of their treatment regimens, immunological or virological parameters. Informed consent was obtained from the study participants and the controls. Patients were excluded only if they refused or were otherwise unable to give consent.

The study participants were recruited from both the public and private sector including the Helen Joseph Hospital (Johannesburg, South Africa), the Chris Hani Baragwanath Hospital (Johannesburg, South Africa), the Charlotte Maxeke Johannesburg Academic Hospital (Johannesburg, South Africa) and the Donald Gordon Medical Centre (Johannesburg, South Africa).

Demographic and clinical information were collected, including the presence of comorbid diseases, drug history, social habits, the presence of chronic infections, the underlying cause for ESKD and the ART regimen. All available (14 of 17 participants) CD4+ T-cell counts and HIV viral loads were documented.

Prior to taking blood samples, the study participants were matched 1:1 with HIV-uninfected patients having ESKD receiving chronic haemodialysis. The control group was selected at each site where the study participants were selected. Controls were selected based on the criteria needed to match them with the HIV-infected group. They were matched with the HIV-infected group for age, sex and body mass index (BMI).

Vascular access was established immediately prior to haemodialysis. Peripheral whole blood samples were collected with a needle and a syringe and placed in a 4.5 mL EDTA tube. Haemodialysis was initiated and continued for 4 hours. A second whole blood sample was collected with a needle and syringe within 10 min after the end of dialysis and placed in a 4.5 mL EDTA tube. The samples were transported at room temperature to the laboratory within 24 h of collection.

All CD4+ T-cell counts were analysed by flow cytometry. Briefly, 100 µL of whole blood was incubated for 10 min in an automated T-Q-Prep machine (Beckman Coulter, Berea, CA, USA) with 5 µL Cyto stat tetra CHROMETM CD45 (fluorescein isothiocyanate (FITC))/CD4(RD1)/CD8(ECD)/CD3 (PC-5) monoclonal antibody (Beckman Coulter Ireland Inc). During the incubation period, a stabiliser, lysing agent and fixative were added. Flow count beads of 100 µL (Beckman Coulter) were then added to the lysate and analysed on a Beckman-Coulter FC500-MPL flow cytometer on a 4-colour T-cell protocol. Absolute T-cell numbers were then calculated using the total white cell count (WCC), and the percentage of lymphocytes and the percentage of CD3 or CD4 or CD8 cells were also calculated and expressed as both an absolute number (cells/µL) and a percentage of WCC.^18^ The CD4+ T-cell count was compared using the laboratory-determined reference range. In four study participants, only CD4+ T-cell counts could be performed.

A normality test (D’Agostino & Pearson normality test) was applied to the data set, and all continuous variables (including the CD4+ T-cell count) were expressed as a median and interquartile range. Comparisons between pre- and post-dialysis parameters were performed using a paired student’s *t* test.

The longitudinal trend analysis of the absolute CD4 counts, the percentage of CD4 cells and the viral loads were analysed using a time series where possible.

All the statistical data were analysed using Graph Pad Prism 7.05. A *p-*value of < 0.05 was considered significant for these analyses.

### Ethical consideration

Ethical approval was obtained from the Human Research Ethics Committee of the University of the Witwatersrand (reference number: M170858).

## Results

A total of 17 participants and 17 controls were included in this study. The controls were matched for age, sex and BMI to the participants. All the study participants were diagnosed with ESKD and were receiving RRT by means of chronic haemodialysis (three sessions per week, and each session lasting ~4 hours).

Renal biopsies had not been performed in most participants (2 of 17; 11%); and in the majority of cases (15 of 17; 88%), the cause of renal failure was inferred from the patient’s medical records. The most common cause for ESKD was stated as hypertension (82%). Most of the study participants had uncontrolled hypertension. Two patients had (renal biopsy confirmed) HIV-associated nephropathy (2%) and one patient had renal failure as a result of ethylene glycol overdose (1%).

All HIV-infected patients were treated with first-line ART regimen at doses adjusted for kidney failure. All HIV-infected participants had received a GeneXpert (Cepheid, Sunnyvale) test for *Mycobacterium tuberculosis* prior to the commencement of haemodialysis. Only a single patient had hepatitis B virus co-infection. The socio-demographic details are summarised in Table 2.

**TABLE 2:** Socio-demographic and categorical variables of the study participants.

Socio-demographics	Study participants (*n* = 17)				Control group (*n* = 17)			
	Median	IQR	*n*	%	Median	IQR	*n*	%
**Age in years**	38	35–42	-	-	38	35–42	-	-
**BMI**	25	21–25	-	-	25	21–25	-	-
**Duration of ART treatment years**	5	3–5	-	-	0	0	-	-
**Duration of haemodialysis in years**	3	3–4	-	-	6	3–6	-	-
**Sex**									
Male	-	-	9	53	-	-	9	53
Female	-	-	8	47	-	-	8	47
**Comorbidities**									
Hypertension	-	-	17	100	-	-	16	94
Diabetes mellitus	-	-	0	0	-	-	1	6
**Social history**									
Reported smoking	-	-	0	0	-	-	0	0
Reported alcohol use	-	-	0	0	-	-	0	0
Reported recreational drug use	-	-	0	0	-	-	0	0
**Chronic infections**									
*Mycobacterium tuberculosis*	-	-	0	0	-	-	0	0
Hepatitis B	-	-	1	0.1	-	-	0	0
**Cause for renal failure**									
Hypertension	-	-	14	82	-	-	16	94
Diabetes mellitus	-	-	0	0	-	-	1	6
HIVAN	-	-	2	12	-	-	0	0
Other	-	-	1	0.1	-	-	0	0

BMI, body mass index; ART, antiretroviral therapy; HIVAN, HIV-associated nephropathy; IQR, interquartile range.

Leucocyte count and T-cell subsets were measured immediately before and after a single session of haemodialysis for the study controls and the study participants. These results are summarised in Tables 3 and 4.

**TABLE 3:** Measured parameters of the study controls.

Variables	WCC (× 10^9/L)		% of CD3+ cells lymphocytes		Absolute CD3 count (cells/µL)		% of CD4+Tcells		Absolute CD4 count (cells/µL)		% of CD8+ T cells		Absolute CD8 cells (cells/µL)	
	Pre-dialysis	Post-dialysis	Pre-dialysis	Post-dialysis	Pre-dialysis	Post-dialysis	Pre-dialysis	Post-dialysis	Pre-dialysis	Post-dialysis	Pre-dialysis	Post-dialysis	Pre-dialysis	Post-dialysis
1	4.07	4.33	79.8	80.7	990	1011	29.4	30.5	291	309	38.8	39.3	384	397
2	3.71	4.57	81.1	82.1	1184	1395	43.5	50	515	698	20.2	18.4	239	257
3	3.51	3.46	72.5	74.3	403	1023	39.6	44.6	404	456	15.4	16.3	157	167
4	3.21	3.68	83.5	83.7	702	619	45.9	47.8	322	296	23.4	20.9	170	129
5	8.20	5.56	69.4	74.7	1983	1930	43.4	48.8	861	942	24.4	23.0	483	444
6	1.51	1.87	67.5	74.0	678	592	35.7	37.1	242	220	31.5	28.1	214	167
7	4.76	4.12	75.6	75.6	1280	1125	44.7	47.7	573	537	35.7	34.9	457	393
*8	5.06	3.80	-	-	-	-	36.2	41.6	424	476	-	-	-	-
*9	3.18	3.23	-	-	-	-	44.6	44.7	432	442	-	-	-	-
*10	5.55	5.41	-	-	-	-	46.4	54.7	465	498	-	-	-	-
*11	3.74	4.33	-	-	-	-	46.7	53.3	236	363	-	-	-	-
12	3.93	4.87	75.6	79.9	977	806	34.5	52.9	337	427	30.1	23.2	294	187
13	4.70	4.63	78.8	80.3	606	564	35.9	47.1	218	265	23.6	21.1	143	119
14	5.32	5.18	71.5	81.1	1290	743	51.1	53.2	659	395	23.2	22.0	299	163
15	3.61	3.75	74.1	73.8	844	904	45.2	45.5	382	412	15.9	19.7	134	178
16	5.01	4.16	71.9	77.3	587	1652	31.3	52.5	268	868	21.8	22.0	272	364
**17**	2.78	2.02	76.5	73.2	779	490	44.8	50.4	349	247	23.9	21.6	186	106
**Mean**	**4.24**	**4.05**	**76.9**	**75.7**	**946**	**998**	**41.1**	**47.2**	**410**	**461**	**25.2**	**23.9**	**264**	**236**
**SD**	**1.50**	**1.03**	**5.7**	**5.7**	**416**	**441**	**6.2**	**6.3**	**168**	**204**	**7.01**	**6.5**	**116**	**120**

WCC, white cell count.

**TABLE 4:** Measured parameters of the study participants.†

Variables	WCC (× 10^9/L)		% of CD3+ cells lymphocytes		Absolute CD3 count (cells/µL)		% of CD4+ T cells		Absolute CD4 count (cells/µL)		% of CD8+ T cells		Absolute CD8 cells (cells/µL)	
	Pre-dialysis	Post-dialysis	Pre-dialysis	Post-dialysis	Pre-dialysis	Post-dialysis	Pre-dialysis	Post-dialysis	Pre-dialysis	Post-dialysis	Pre-dialysis	Post-dialysis	Pre-dialysis	Post-dialysis
1	5.24	4.43	78.4	83.5	575	652	24.7	29.6	181	231	50.4	51.1	370	399
2	2.74	2.85	82.3	83.6	546	777	31.5	37.2	209	346	49.6	44.9	330	417
3	5.11	3.6	73.3	75.6	1174	720	24.1	29.8	387	284	48.4	44.5	778	424
4	5.38	3.96	84.4	86.5	740	813	39.3	40	345	376	44.2	43.7	388	411
5	6.52	5.86	71.5	75.7	609	584	38.0	47.4	324	366	31.4	27.3	267	211
6	6.97	6.47	73.1	79.5	1009	690	10.0	15.2	139	132	57.5	58.8	794	511
7	4.12	6.88	78.5	78.0	777	1400	40.8	43.7	404	785	34.8	31.9	345	574
†8	2.94	2.95	-	-	-	-	29.8	32.7	227	281	-	-	-	-
†9	3.95	3.94	-	-	-	-	25.0	29.6	260	328	-	-	-	-
†10	3.25	3.80	-	-	-	-	38.4	44.2	414	521	-	-	-	-
†11	5.30	6.07	-	-	-	-	28.2	36.5	222	330	-	-	-	-
12	6.88	6.63	78.8	82.9	1905	1734	38.4	46.6	929	975	37.2	33.3	901	697
13	3.65	4.2	82.1	83.5	834	980	27	36.4	274	428	51.8	43.9	526	515
14	5.86	5.17	79.1	87.5	1576	1270	44.5	54	888	784	27.0	27.1	538	393
15	3.63	3.07	75.2	75.5	766	765	33.8	34.4	349	357	40.3	39.4	416	408
16	5.62	5.26	73.4	77.3	770	870	44.6	50.4	483	564	27.3	26.9	287	301
**17**	7.11	7.03	78.3	78.6	1390	1294	29.8	30.0	538	544	46.7	43.2	843	750
**Mean**	**4.69**	**4.83**	**77.5**	**80.2**	**974**	**965**	**32.23**	**37.51**	**386.6**	**448.9**	**42.05**	**39.69**	**521.8**	**462.4**
**SD**	**1.81**	**1.44**	**4.01**	**4.6**	**420**	**350**	**8.9**	**9.6**	**223.9**	**227**	**9.8**	**9.87**	**228.6**	**±147.9**

WCC, white cell count.

† , CD8%, CD3% and absolute counts not collected.

For the HIV-uninfected study controls, the following pre-dialysis parameters were less than the normal reference ranges: total leucocyte count (5.9%), absolute CD4+ T-cell count (29%) and the absolute CD8+ T-cell count (23%). In addition, the following post-dialysis parameters were less than the normal reference ranges used: absolute CD4+ T-cell count (23%) and the absolute CD8+ T-cell count (38%; Table 3).

All the study participants’ total leukocyte counts, absolute CD3+ T-cell counts and the absolute CD8+ T-cell counts were within the normal laboratory reference range for adults. The pre-dialysis absolute CD4+ T-cell count was lower than the normal laboratory reference range in eight HIV-infected patients (47%), and two patients had an absolute CD4+ T-cell count of < 200 cells/µL. The post-dialysis absolute CD4+ T-cell counts were lower than the normal reference range in six (35%) of the HIV-infected patients. Only one patient presented with an absolute CD4+ T-cell count of < 200 cells/µL (Table 4). See Table 5 for reference ranges.

**TABLE 5:** Internally established laboratory reference ranges for leucocyte count, percentage and absolute lymphocyte subsets.

Variable	WCC (× 10^9/L)	CD3+ T-cell count (cells/µL)	% CD3+ T cells	CD4+ T-cell count (cells/µL)	% CD4+ T cells	CD8+ T-cell count (cells/µL)	% CD8+ T cells
Reference range	2.5–10.40	527–2846	59–81	332–1642	28–51	170–811	12–38

WCC, white cell count.

**FIGURE 1:**
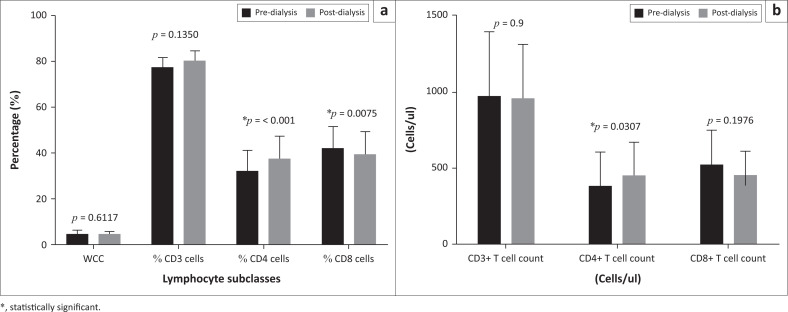
Measured parameters of study participants pre- and post-dialysis.

For the study participants, no statistically significant change was observed in the total leukocyte count (*t* = 0.5178, *p* = 0.612), the T-cells (CD3+ cells) as a percentage of the lymphocyte count. (*t* = 1.609, *p* = 0.142) or the absolute CD3+ T-cell count (*t* = 0.122, *p* = 0.901) after haemodialysis. A statistically significant increase was noted in the post-dialysis CD4+ T cells as a percentage of lymphocytes (*t* = 7.106, *p* = 0.001) as well as the absolute CD4+ T-cell count counts (*t* = 2.371, *p* = 0.032). A statistically significant decrease in the post-dialysis percentage of the CD8+ T cells (*t* = 3.212, *p* = 0.008) was found (Figure 1).

### Figure 1: Measured parameters of study participants pre- and post-dialysis

**FIGURE 2:**
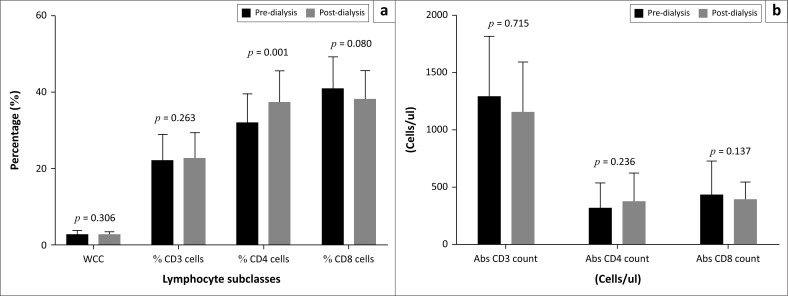
Measured parameters of study controls pre- and post-dialysis

For the study controls, the percentage of CD4+ T cells was the only immunological parameter to show a statistically significant increase after haemodialysis (*t* = 4.195, *p* = 0.001). The other measured parameters revealed no statistically significant change (Figure 2).

### Figure 2: Measured parameters of study controls pre- and post-dialysis

Following a cross-sectional analysis, the longitudinal trends for the percentage of CD4+ T cells, the absolute CD4+ T-cell counts and the HIV viral loads were analysed for each patient. The initial available CD4+ T-cell count was taken as point zero. Although the exact date when haemodialysis was started for each patient is not certain, it is known that point zero was obtained prior to haemodialysis initiation. During this period, it is not certain whether all the participants were on ART and the compliance of the patients to their treatment.

Five HIV-infected study participants (patients 1, 4, 7, 10 and 11) showed a statistically significant longitudinal increase in the percentage of CD4+ T cells and patient 8 showed a trend towards increased percentage of CD4+ T cell which was not significant (Appendix 1).

Two HIV-infected study participants (patients 9 and 11) had a statistically significant decline in their absolute CD4+ T-cell counts. Eight of the patients showed a statistically non-significant rise in their absolute CD4+ T-cell counts, and four patients had a statistically non-significant decline in their absolute CD4+ T-cell counts (Appendix 1).

Virological suppression is a pre-requisite for the deceased donor kidney transplantation. Although all HIV-infected patients were receiving the standard first-line ART, only three study participants showed virological suppression below the level of viral load detectability as performed in our lab. The cross-sectional median viral load was 44 500 copies/mL (± 9753.4 – 51 698.04). In patients for whom longitudinal data were available (14/17), most patients displayed a stable viral load (*n* = 13). Only one patient (patient 6) had a statistically significant increase in the HIV viral load (Appendix 1).

## Discussion and conclusion

Kidney transplantation is a cost-effective and curative strategy in patients with ESKD irrespective of having HIV infection or not. Eligibility criteria could, however, limit access to this treatment particularly if these could be impacted by RRT. A CD4+ T-cell count above 200 cells/mL is a pre-requisite for deceased donor kidney transplantation in South Africa.^16^ This study investigated whether alternative forms of RRT, specifically haemodialysis, had an immediate on the lymphocyte subsets in PLWH with ESKD.

The CD4+ T-cell count as a percentage of lymphocytes and as an absolute number increased immediately following haemodialysis in both HIV-infected participants and uninfected controls. These findings contradict previously published data which suggest that absolute CD4+ T-cell counts decline immediately post-dialysis.^12^ The apparent increase in the CD4+ T cells may reflect the loss of CD8+ T cells and concomitant haemo-concentration. The CD4+ T cells may also have been recruited from other areas such as solid lymphoid tissue. The effector functions of these cells are uncertain. Importantly no decrease was reported in CD4+ T cells in the immediate post-dialysis period. No patients developed a CD3+ T-cell lymphopenia or a decreased CD8+ T-cell count before dialysis although HIV-infected patients had significantly lower CD4+ T-cell counts prior to dialysis than uninfected controls.

Only the percentage (%) of CD8+ T cells showed a significant decrease post-haemodialysis in the HIV-infected study participants. However, the absolute CD8+ T-cell counts did not show a statistically significant decline post-dialysis. This contrasts with previous studies in HIV-uninfected patients with ESKD receiving haemodialysis, revealed lower levels of absolute CD8+ T cells when compared to normal controls.^19,20,21^ The decrease in the CD8+ T-cell count is postulated to be caused by activation of these cells by the dialyser membrane with subsequent apoptosis of these cells.^19,22^

We went on to assess the longitudinal trends of percentage of CD4+ T cells, absolute CD4+ T cells and the HIV viral loads based on retrospective laboratory data for 14 of our study participants. The majority of our population showed a stable (*n* = 8) or increased CD4+ T-cell count over time (*n* = 5) above 200 cells/µL. According to the national guidelines for renal transplantation in PLWH, this is the minimum absolute CD4+ T-cell count required for listing deceased donor transplantation.^16^ Ongoing investigations are being conducted to establish the optimal absolute CD4+ T-cell count for the best possible outcome. It appears that an absolute CD4+ T-cell count of 200 cells/µL may be inadequate to protect against adverse outcomes including post-transplant opportunistic infections.^17^ A study conducted in our setting evaluating the longitudinal trends of PLWH with ESKD on chronic haemodialysis found an annual increase in the longitudinal absolute CD4+ T-cell counts in PLWH with ESKD on chronic haemodialysis.^23^

The frequency of HIV viral load testing performed varied amongst the different centres treating these patients. The current national HIV treatment guidelines state that the immunological (CD4+ T-cell count) and virological (HIV viral load) parameters should be measured at initially 6- then 12-monthly intervals in PLWH.^24^ The majority of our patients were not virologically suppressed despite this being an eligibility criterion for deceased donor kidney transplantation. This, however, is not an uncommon finding in PLWH with ESKD on chronic haemodialysis. Studies assessing the longitudinal HIV viral loads in PLWH having ESKD on chronic renal dialysis found approximately half of their cohort of patients did not have a suppressed HIV viral load.^23,25^ Possible reasons include haemodialysis, itself, which may lead to an increase in HIV replication because of the release of specific cytokines as well as the use of certain dialysis membranes during the haemodialysis procedure.^26,27^ Other possible causes include unreliable adherence to ART, inexperience with prescribing ART (suboptimal dosing as a result of the renal failure), infrequent consultations with infectious diseases specialists, patient compliance and ART timing (before or after haemodialysis) which in turn could influence drug concentrations.^23^

This study has numerous limitations. Firstly, the number of PLWH having ESKD currently receiving chronic haemodialysis in four different academic centres in Johannesburg is small. It is likely that the small number reflects the strict qualification criteria for dialysis and the limited dialysis slots available. Secondly, the selection of the study participants was not randomised and selection bias cannot be excluded in this cohort of patients. In addition, the exact date when haemodialysis was started for each patient is not certain, and it is also not certain whether all the participants were on ART and the compliance of the patients to their treatment; the data on longitudinal CD4+ T-cell counts and viral loads were not always available and the timing of testing were inconsistent with respect to dialysis sessions although the longitudinal trend appears to support the peri-dialysis cross-sectional data. For the same reason, it is not possible to assess the correlations between the T-cell profiles with the viral load before and after the start of dialysis. It was not possible in this small study to perform ART monitoring to ensure that the absence of virological suppression did not reflect the lack of adherence. Unfortunately, a control group could not be added to the longitudinal analyses of the study participants. As the control group comprised HIV-uninfected individuals, routine CD4+ T cell testing is not performed in these patients.

This study failed to show a negative effect of haemodialysis on the CD4+ T-cell count. However, unexpectedly, the absolute CD4+ T-cell count increases immediately post-dialysis, suggesting that immunologic recovery is not impeded by the treatment of the underlying ESKD. Further studies are required to ascertain the possible reasons for a rise, how long this rise is sustained and whether these CD4+ T cells are functional. Of concern, the patients in this study failed to show virological suppression; because this is a key driver of disease progression and complications including non-communicable diseases, this requires urgent investigation.

## Appendix 1

**TABLE 1-A1:** Time series analysis of the longitudinal absolute CD4+ T cell count, %CD4+ T cells and HIV viral loads (VL).

Patient		Year 1	Year 2	Year 3	Year 4	Year 5	Year 6	*R*^2^ value	*P* value
1	Abs CD4	66.00	129.00	226.00	256	210.5	183.30	0.430	< 0.3000
	%CD4	10.20	14.29	20.70	17.29	21.05	27.41	0.850	< 0.0005
	HIV VL	-	70.00	79.50	336.00	135.50	und	0.010	< 0.9100
2	Abs CD4	720.00	469.00	600.00	648.50	647.00	404.50	0.200	< 0.2000
	%CD4	27.36	20.30	25.00	25.78	23.48	30.34	0.160	< 0.0520
	HIV VL	29.00	85.00	33.00	502.00	426.00	und	0.100	< 0.3600
3	Abs CD4	208.00	362.00	343.00	352.50	335.50	-	0.370	< 0.2000
	%CD4	10.75	35.52	36.61	33.03	26.95	-	0.200	< 0.4000
	HIV VL	-	449.00	916.40	82.30	37.50	-	0.430	< 0.3100
4	Abs CD4	320.00	570.00	402.00	415.00	390.00	-	0.001	< 0.7000
	%CD4	24.78	26.63	30.67	34.31	38.47	-	0.980	< 0.0010
	HIV VL	182.50	600.25	300.50	554.67	111.00	-	0.020	< 0.9300
5	Abs CD4	218.00	313.67	-	-	-	-	-	< 0.3000
	%CD4	31.52	41.30	-	-	-	-	-	< 0.3000
	HIV VL	50.00	905.50	und	-	-	-	-	< 0.9700
6	Abs CD4	129.50	29.00	177.00	129.30	-	-	0.090	< 0.3000
	%CD4	17.64	3.35	9.27	12.15	-	-	0.050	< 0.3000
	HIV VL	und	161255.00	344.00	522500.00	-	-	0.500	< 0.0020
7	Abs CD4	506.50	575.00	528.00	-	-	-	-	< 0.9900
	%CD4	31.32	36.20	41.25	-	-	-	-	< 0.0097
	HIV VL	377.00	151.00	und	-	-	-	-	< 0.2200
8	Abs CD4	272.60	374.50	-	-	-	-	-	< 0.0900
	%CD4	24.90	24.10	-	-	-	-	-	< 0.4000
	HIV VL	201765.00	und	-	-	-	-	-	< 0.0600
9	Abs CD4	307.00	316.50	228.70	250.00	-	-	0.600	< 0.2000
	%CD4	10.49	21.53	20.63	20.08	-	-	0.490	< 0.5500
	HIV VL	88353.00	27.50	115500.00	9601.00	1061.00	-	0.230	< 0.1900
10	Abs CD4	276.00	234.00	453.00	423.50	461.70	323.50	0.040	< 0.9000
	%CD4	23.74	20.56	26.00	28.13	35.10	30.78	0.700	< 0.0200
	HIV VL	und	und	und	und	und	763.00	0.600	< 0.2700
11	Abs CD4	440.50	952.00	-	-	-	-	-	< 0.0070
	%CD4	39.70	42.50	-	-	-	-	-	< 0.0025
	HIV VL	und	und	-	-	-	-	-	-
12	Abs CD4	440.50	952.00	-	-	-	-	-	< 0.3000
	%CD4	39.70	42.50	-	-	-	-	-	< 0.5100
	HIV VL	und	23.00	-	-	-	-	-	< 0.1700
13	Abs CD4	689.00	486.00	351.00	-	-	-	-	< 0.0800
	%CD4	33.31	32.00	31.70	-	-	-	-	< 0.8000
	HIV VL	und	7170.00	-	-	-	-	-	> 0.9900
14	Abs CD4	982.50	605.50	724.80	-	-	-	-	< 0.3000
	%CD4	41.05	40.47	47.37	-	-	-	-	< 0.0900
	HIV VL	und	und	-	-	-	-	-	< 0.1700
